# An Indirect Measurement Methodology to Identify Load Fluctuations on Axial Turbine Runner Blades

**DOI:** 10.3390/s20247220

**Published:** 2020-12-16

**Authors:** Arash Soltani Dehkharqani, Fredrik Engström, Jan-Olov Aidanpää, Michel J. Cervantes

**Affiliations:** 1 Division of Fluid and Experimental Mechanics, Luleå University of Technology, SE-971 87 Luleå, Sweden; fredrik.1.engstrom@ltu.se (F.E.); Michel.Cervantes@ltu.se (M.J.C.); 2 Division of Product and Production Development, Luleå University of Technology, SE-971 87 Luleå, Sweden; jan-olov.aidanpaa@ltu.se

**Keywords:** prototype Kaplan turbine, load fluctuation on the runner, pressure measurement, strain measurement, axial strain, torsion strain, bending strain, indirect measurement

## Abstract

Smooth integration of intermittent energy sources, such as solar and wind power, into the electrical grid induces new operating conditions of the hydraulic turbine by increasing the off-design operations, start/stops, and load variations. Therefore, hydraulic turbines are subject to unstable flow conditions and unfavorable load fluctuations. Predicting load fluctuations on the runner using indirect measurements can allow for optimized operations of the turbine units, increase turbine refurbishment time intervals, and avoid structural failures in extreme cases. This paper investigates an experimental methodology to assess and predict the flow condition and load fluctuations on a Kaplan turbine runner at several steady-state operations by performing measurements on the shaft in the rotating and stationary frame of references. This unit is instrumented with several transducers such as miniature pressure transducers, strain gages, and proximity probes. The results show that for any propeller curve of a Kaplan turbine, the guide vane opening corresponding to the minimum pressure and strain fluctuations on the runner blade can be obtained by axial, torsion, and bending measurements on the shaft. Torsion measurements on the shaft could support index-testing in Kaplan turbines particularly for updating the cam-curve during the unit operation. Furthermore, a signature of every phenomenon observed on the runner blade signals, e.g., runner frequency, rotating vortex rope components, and rotor-stator interaction, is found in the data obtained from the shaft.

## 1. Introduction

The significant increase in the installation of renewable power generation capacity continued globally over years and maintained more than 8% average growth in the previous five years to reach 2588 GW in 2019 [1]. More than 85% of the additional installed capacity was solar and wind power in 2019 [1]. On one hand, this share of intermittent energy sources directly influences the hydropower generation and demands more flexible operations of hydropower under transient operations to stabilize the electrical grid. On the other hand, hydropower still provides most of the renewable electricity production in the world. Therefore, hydraulic turbines are considered as one of the key players in the electricity market that have to undergo increasing off-design operations with large load fluctuations.

Condition monitoring and safe operation of hydraulic turbines are ensured with monitoring systems that actively monitor and protect the turbine’s health. Without a reliable health monitoring system, the turbine may encounter severe issues, and in a worst-case scenario, a catastrophic failure can occur. Condition monitoring can also be used for life expectancy determination and cost estimation of start/stop cycles. Online monitoring of shaft and bearings vibration, as well as shaft displacement, are methods used to identify undesirable conditions in turbine operation and avoid high load fluctuations on the turbine structure [2,3]. Runner blades of a hydraulic turbine are one of the most vulnerable parts of reaction turbines. They can experience large pressure fluctuation, fatigue, erosion, and cavitation, particularly close to the trailing edge [4,5,6,7]. This can change the natural frequency of the runner blade and damage the blade [8]. In addition, frequent load variations may induce fatigue issues in the Kaplan turbine’s shaft due to runner blade movement [9]. Runner blades are subjected to two types of loading; static loading due to the turbine head and discharge passing through the turbine and dynamic loading produced by different dynamic phenomena such as rotating vortex rope (RVR), rotor-stator interaction, rotating stall, cavitation, and tip vortex [10]. These dynamic phenomena are influenced by the turbine operating point in which the contribution of each source on the runner dynamic response varies [11]. Therefore, dynamic load prediction on the runner is essential to avoid high-pressure fluctuations and mechanical resonance.

Although experimental measurement on the runner blade of prototype hydraulic turbines is a costly and demanding technique, it is still considered a common method to determine the load fluctuations and strain concentrated locations on the runner blade as well as fatigue life estimation [12,13,14]. Recent investigations focused on predicting the flow phenomena in the turbine chamber without performing a direct measurement on the runner blades. An experimental study on a 444 MW prototype Francis turbine suggested that the accelerometers located in the turbine guide vane, bearing, head cover, and in the shaft captured more phenomena compared to other sensors installed in other locations of the turbine [15]. Strain measurements on the shaft of a propeller turbine during start-up operations showed that torsion measurement can be correlated to the strain measured on the blade hotspot and be used for turbine start-up optimization [16]. Another experimental investigation on a 200 MW prototype Francis turbine showed that shaft oscillation at the lower guide bearings increased at a part load operation when the pressure fluctuations were intense in the draft tube [17]. However, there was no correlation found between the shaft oscillations and pressure fluctuation in the draft tube.

Aiming at providing deeper insight into the prototype hydraulic turbines, various operating conditions such as part load operation, load increase, load decrease, startup, speed-no-load, generator excitation, and ramp-up operating conditions have been previously investigated on a Kaplan prototype turbine [18,19,20]. Earlier experimental works on prototype and model hydraulic turbines indicated that a signature of hydraulic phenomena in the turbine can be detected by pressure measurement on the draft tube wall or strain measurement on the shaft [15,20,21]. However, any comprehensive investigation on the correlation between the load fluctuation on the runner and either the draft tube pressure fluctuation or shaft strain and displacement measurement has not been published. The primary goal of this study is to investigate the interaction of the flow and turbine runner blades as well as the turbine shaft in a Kaplan prototype turbine, the Porjus U9 turbine, at different steady-state operating points, on- and off-cam. Pressure and strain measurements on a runner blade as well as strain and displacement measurements on the shaft were performed. Based on the experimental data, time series and spectral analysis are carried out and the influence of the flow on the runner blade and shaft is investigated. Possible correlations between the measurements are investigated, highlighting the potential of shaft measurements to assess the turbine optimum operating conditions and flow phenomena acting on the runner.

## 2. Case Study

### 2.1. Experimental Set-Up

The test case is a 10 MW Kaplan turbine, the Porjus U9 turbine, located in Porjus, Sweden. This turbine is part of the Porjus Hydropower center and is mainly used for research, development, and training purposes. This turbine is featuring 6 runner blades, 20 equally spaced guide vanes, and 18 unequally distributed stay vanes with a runner diameter of 1.55 m. The runner is located approximately 7 m below the tailwater. The penstock has a total length of approximately 67.1 m with three elbows. The diameter of the penstock varies between 10.5 m at the inlet and 2 m near the spiral case inlet. The draft tube length is approximately 11 m. The nominal operating parameters of this turbine are presented in Table 1. 

### 2.2. Instrumentation

**Figure 1:**
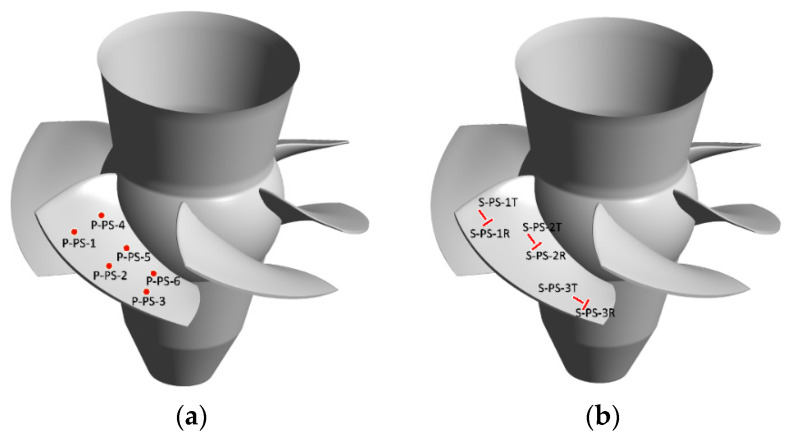

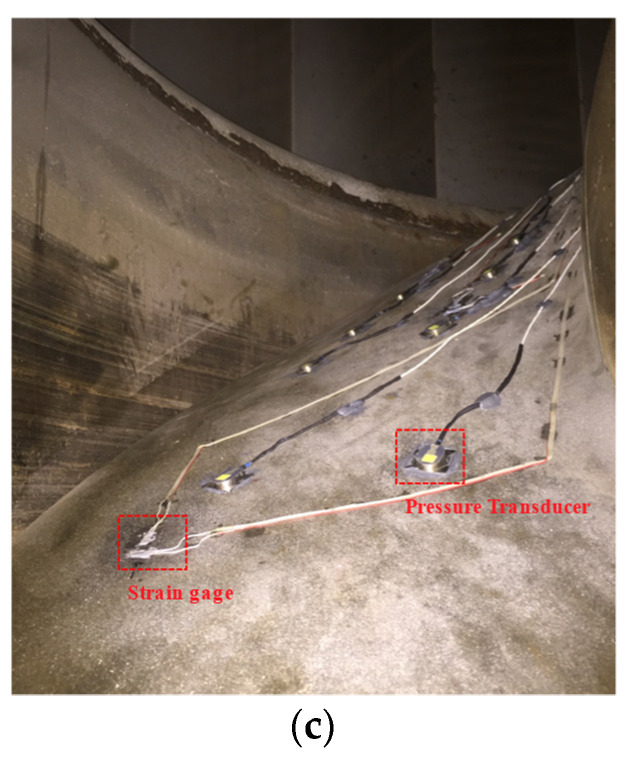
(**a**) Position of the pressure transducers on the runner blade pressure side [20], (**b**) position of the strain gages on the runner blade pressure side [20], (**c**) the blade runner pressure side before applying the epoxy.

**Figure 2:**
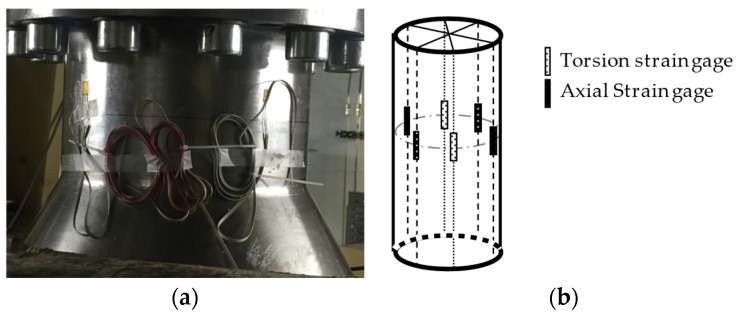
(**a**) Installed strain gages on the turbine shaft, (**b**) arrangement of the strain gages on the turbine shaft [18].

Pressure, strain, and displacement measurements were performed at steady-state operating conditions. A total of twelve miniature piezo-resistive pressure transducers (Kulite, LL-080 series) were placed on a runner blade. Six pressure transducers were installed on the pressure side (P-PS-1 to P-PS-6) and six on the suction side (P-SS-1 to P-SS-6) of one runner blade. The operating range of the pressure transducers is 0–700 kPa, and their natural frequency is 380 kHz. The pressure transducers were located at the intersection of imaginary circles that pass through 1/3 and 2/3 of the blade’s span and 1/4, 1/2, and 3/4 of the blade’s chord lines. Three hotspots were selected on the runner blade to locate strain gages based on finite element calculations. Two uniaxial strain gages (K-LY41-6/350-3-2M manufactures by HBM) were installed at each point in the tangential and radial directions on the blade pressure and suction sides, respectively. Therefore, twelve strain gages were installed on the runner blade. The resistance and gage factor of the strain gages were 350 ± 0.35% and 2.07 ± 1%, respectively. Figure 1 presents the position and arrangement of the pressure transducers and strain gages on a runner blade. Six strain gages were installed on the turbine shaft surface between the generator guide bearing and the turbine guide bearing to measure axial strain, bending strains in two directions, and torsional strain. The axial strain and the bending strain in two directions were measured by four uniaxial strain gages, the same type used for the strain measurements on the blade, installed in the direction of the shaft’s rotating axis with a 90° spacing. The shaft torsional strain measurement was performed using two torsion strain gages (K-XY41-6/350-3-2M manufactures by HBM) installed on the same shaft section in the axis direction with 180° spacing. The resistance and gage factor of the torsional strain gages were 350 ± 0.35% and 2.08 ± 1%, respectively. Figure 2 presents the picture and arrangement of the strain gages on the turbine shaft.

**Figure 3:**
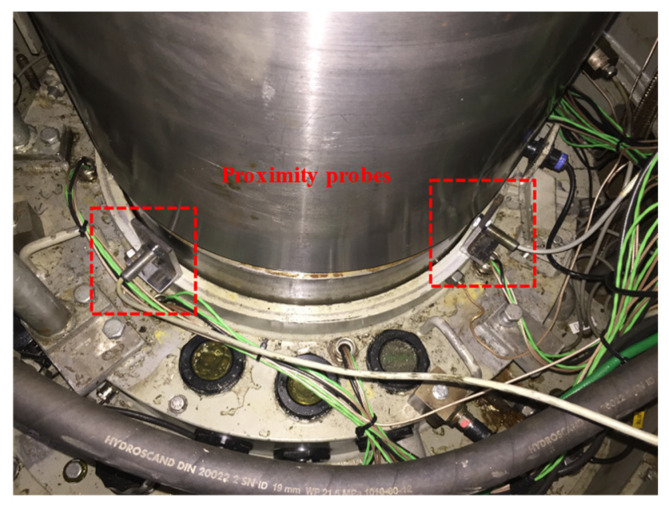
Location of the proximity probes installed close to the turbine guide bearing.

The lateral displacement of the shaft was measured by two proximity probes (DW-AD-509-M12 manufactured by Contrinex) installed close to the turbine guide bearing with a 90° spacing. The sensing range was 0–6 mm. Figure 3 shows the location of the proximity probes installed close to the turbine guide bearing.

Turbine operational parameters such as guide vane opening, runner blade opening angle, power output, headwater level, and tailwater level were simultaneously recorded from the turbine control unit. In addition, the guide vane opening was measured by a distance transducer of wire type installed on the guide vane hydraulic servomotor to acquire the linear movement of the guide vanes. The runner blades pitch angle and the guide vane angular opening range of this unit is +10° to −17° and 0–35° (0–100% opening), respectively. An optical encoder was installed close to the shaft to measure the turbine rotational speed. The accuracy of the optical encoder was 3°.

The uncertainty analysis for different transducers was determined using six nonconsecutive repeated measurements at OP5, which is presented in the next section. The uncertainties of the pressure transducers, strain gages, and proximity probes are presented in Table 2. 

### 2.3. Kaplan Turbine Operation and Measurement Program

Kaplan turbines are classified as double-regulated reaction turbines because the flow conditions are controlled by adjustable guide vanes and runner blades pitch angle to obtain the best hydraulic efficiency at a specific head. Classical index testing identifies the optimal operating point of various propeller curves for a Kaplan turbine. Then, a cam-curve is derived from the index-test assuring a high-efficiency operation at different flow conditions and heads. The turbine operation based on the cam-curve is called on-cam operation in which the guide vanes and runner blades pitch angle are automatically adjusted. However, the runner blades pitch is set to a prescribed angle and held constant at off-cam operation, i.e., the turbine is operated as a single regulated turbine. Under an on-cam condition, turbine operating conditions can be specified at different guide vane opening, e.g., deep part load, part load, best efficiency point (BEP), and high-load. These conditions can also be defined for each propeller curve obtained in the index-test.

**Figure 4:**
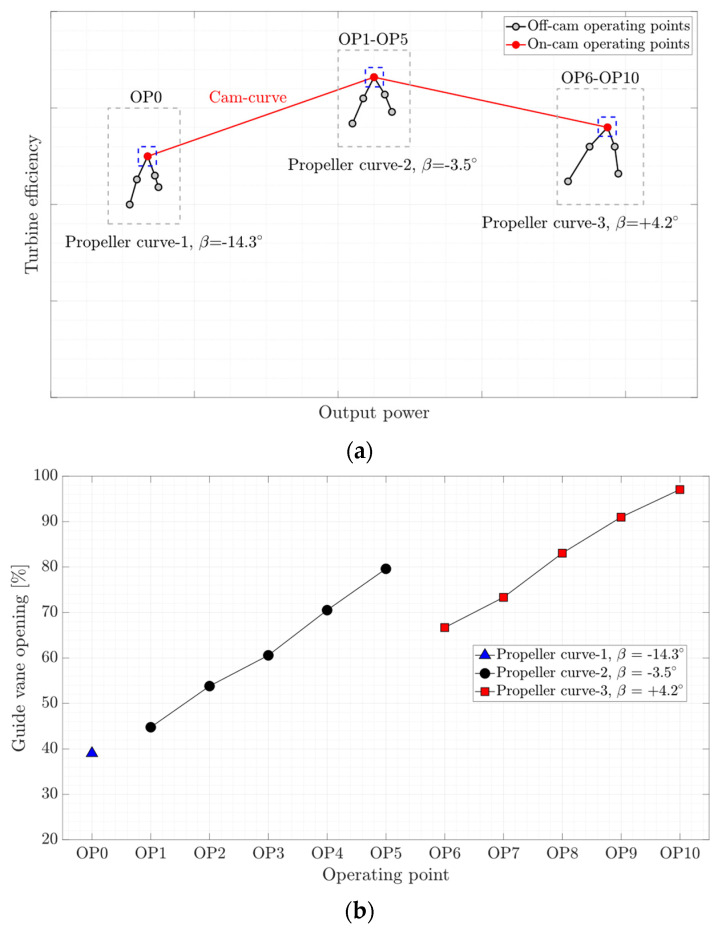
(**a**) Schematic of the investigated operating conditions, (**b**) the guide vane opening and runner blades pitch angle variation for different operating points; ‘OP’ denotes operating point.

The experimental investigation of the turbine was performed at eleven steady-state operating points selected from three propeller curves. Figure 4a presents a schematic of the operating points. The on-cam operating points (OP0, OP3, and OP8), located on the cam-curve (red color) highlighted by the blue dashed-line box, are considered the BEP of the first, second, and third propeller curve, respectively. Looking at the turbine on-cam operation, OP0, OP3, and OP8 are considered as deep part load, BEP, and high-load operating points, respectively. Moreover, eight off-cam operating points were selected from two propeller curves (propeller curve-2 and propeller curve-3) covering part load and high load operation of the unit while operating in off-cam mode. Figure 4b presents the guide vane opening of the investigated operating points. The runner blades pitch angle of the first, second, and third propeller curves is −14.3°, −3.5°, and +4.5°, respectively.

## 3. Analysis

### 3.1. Time Domain Analysis

Averaging the acquired steady-state data was carried out using Equation (1).

(1) $$\bar{X}=\frac{\sum_{i=1}^{k}X_{i}}{k}$$

where k is the total number of samples, and $X_{i}$ is the variable value. The peak-to-peak amplitude is calculated by considering 2σ, where σ is the standard deviation of the quantity and calculated by Equation (2).

(2) $$\sigma =\sqrt{\frac{1}{k-1}\overset{k}{\underset{i=1}{\sum }}|X_{i}-\bar{X}{|}^{2}}$$

### 3.2. Frequency Analysis

Spectral analysis of the data was performed to determine the dominant frequencies of the flow and shaft. The time series of each data were padded around zero by subtracting the time-averaged data obtained from the instantaneous data. Welch’s method with a Hanning window was applied to the calculated fluctuating component. The steady-state data was divided into six segments, and an overlap of 50% was considered between the segments. The frequencies are normalized as

(3) $$f_{n}=\frac{f}{N}$$

where f is the frequency component, and N is the rotational frequency of the runner. The amplitudes obtained from the spectral analysis are normalized as

(4) $$amp=\frac{Y-Y_{min}}{Y_{max}-Y_{min}}$$

where Y is an array of amplitudes, and the subscript min and max denote the minimum and maximum value of the array of amplitudes, respectively.

## 4. Results

Experimental measurements were performed on a prototype Kaplan turbine at eleven steady-state operating points. The results of four pressure transducers installed on a runner blade pressure side (P-PS-2, P-PS3, P-PS-4, and P-PS-6), two strain gages close to the hub (S-SS-5R and S-SS-5T), and two strain gages close to the trailing edge (S-SS-6R and S-SS-6T), as well as axial, torsion, and bending measurements in two directions on the turbine shaft, and shaft displacement are presented. The time-averaged and peak-to-peak amplitudes of the results will be presented. Thereafter, their spectral analysis results will be discussed, and a summary of the phenomena detected by the transducers will be presented.

### 4.1. Time-Averaged Parameters

**Figure 5:**
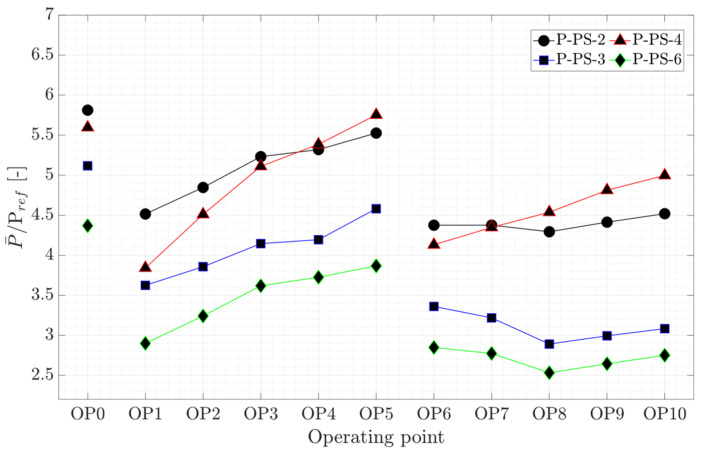
Normalized average pressure of four pressure transducers on the runner blade at OP0–OP10.

In this study, the pressure data is normalized with respect to the initial pressure value in the turbine chamber before running the measurement ($P_{ref}$). The normalized time-averaged pressure variation on the runner blade pressure side for OP0–OP10 is presented in Figure 5. The pressure increases for all the pressure transducers for the operating points OP1 to OP5 (propeller curve-2) as the discharge increases with the guide vanes opening. In the propeller curve-3, similar increasing behavior is observed for the pressure transducer located at the leading edge close to the runner hub (P-PS-4) from OP6 to OP10. However, the pressure magnitude of the other pressure transducers shows a minimum value at OP8, which is considered as the BEP of the propeller curve-3. Despite the lower guide vane opening at OP0, the pressure magnitudes are higher than at other operating points due to the nearly closed runner blades position.

**Figure 6:**
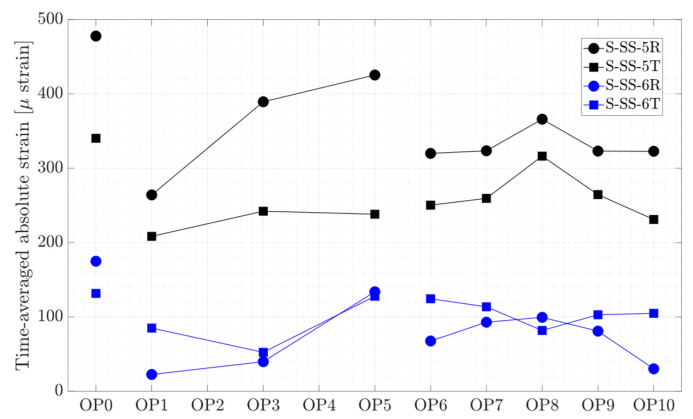
Time-averaged strain obtained on the runner blade.

The time-averaged strain variation on the blade obtained from four strain gages during the steady-state operating conditions is presented in Figure 6. A high strain is observed close to the blade hub compared to other locations. Similar to the relatively high-pressure magnitude at OP0, the strain magnitude is also higher compared to other operating conditions. The variation of the strain on the blade in the radial and tangential direction for each propeller curve is different. This shows that the discharge is not the only parameter affecting the strain on the blade.

**Figure 7:**
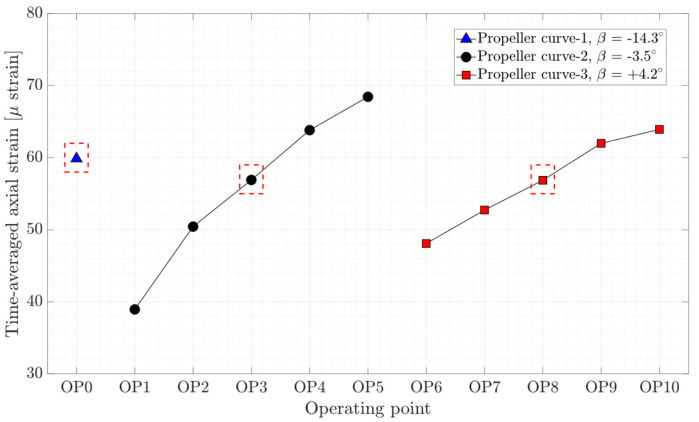
Time-averaged axial strain obtained on the shaft at OP0–OP10; red dashed-line boxes indicate the on-cam operating points.

**Figure 8:**
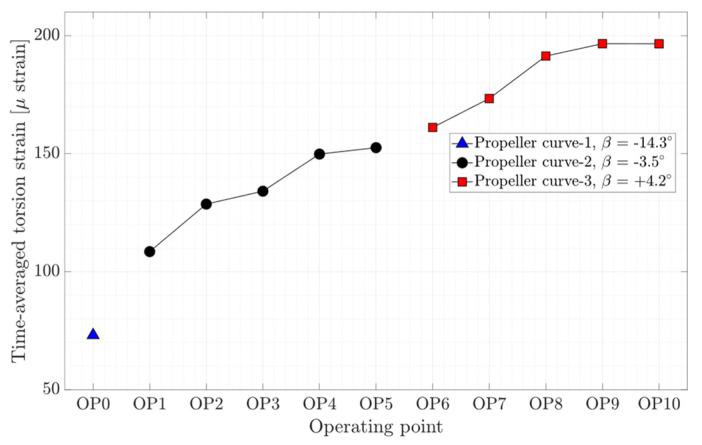
Time-averaged torsion strain obtained on the shaft at OP0–OP10.

**Figure 9:**
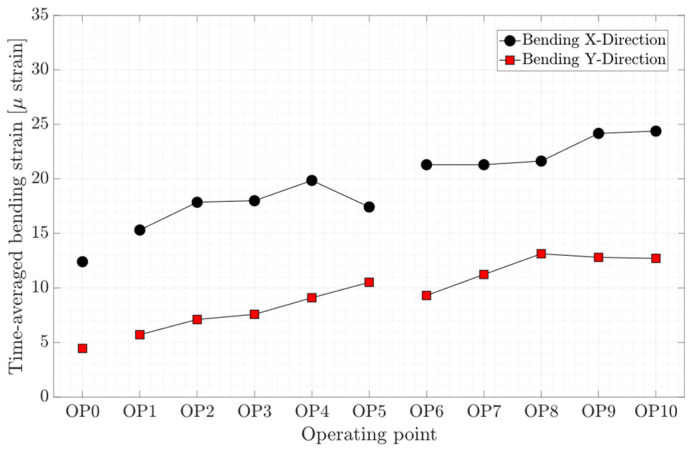
Time-averaged bending strain obtained on the shaft at OP0–OP10.

The time-averaged axial, torsion, and bending strain obtained on the shaft for different operating conditions are presented in Figure 7, Figure 8, and Figure 9, respectively. As expected, the axial and torsion strain increase with the guide vanes opening for the propeller curve-2 and propeller curve-3, meaning from OP1 to OP5 and OP6 to OP10. Interestingly, the axial strain on the shaft at different on-cam operating points, which are highlighted by red dashed-line boxes, is rather constant, although different guide vane openings and runner blades pitch angles were assigned to the operating points. Like axial strain variation, the bending strain in the X and Y direction increases with the guide vanes opening except for the bending strain in the X direction at OP5 and bending strain in the Y-direction at OP9 and OP10. Despite the deep part load operation at OP0, the bending strain in both directions is lower than other operating points.

### 4.2. Peak-to-Peak Amplitudes

**Figure 10:**
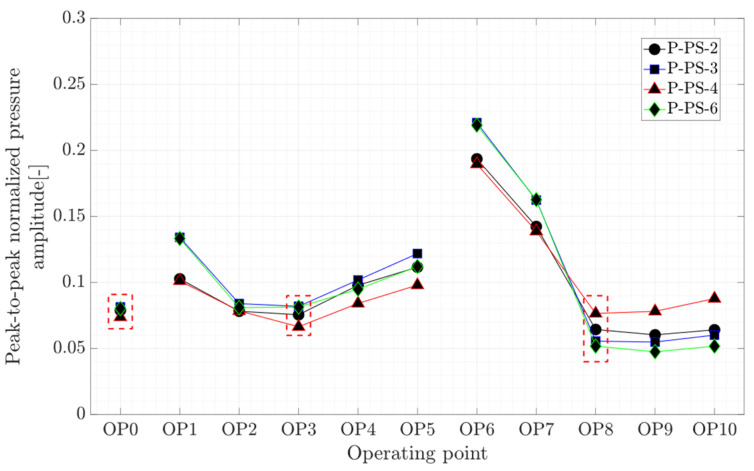
Peak-to-peak normalized pressure amplitude of four pressure transducers on the runner blade at OP0–OP10; red dashed-line boxes indicate the on-cam operating points.

From the fatigue and dynamics points of view, load fluctuations on the runner blade of hydraulic turbines are more important than absolute magnitudes. In this section, peak-to-peak amplitudes on the runner blade and shaft are calculated and compared. Figure 10 presents the peak-to-peak normalized pressure amplitude obtained on the runner blade at the steady-state operating points. The trend of the peak-to-peak variation for the propeller curve-2 and propeller curve-3 is different. In the propeller curve-2, OP1 to OP5, the peak-to-peak variation is rather symmetric with respect to its BEP (OP3). However, in the propeller curve-3 with a larger runner blades pitch angle (+4.2°), large peak-to-peak pressure amplitudes are observed at OP6 and OP7 compared to OP9 and OP10, indicating the presence of large pressure pulsation arising from the RVR. Later in the spectral analysis of pressure transducers, it is shown that the amplitude of RVR components at OP6 and OP7 is higher compared to other part load operating points, i.e., OP1 and OP2, located on the propeller curve-2. The peak-to-peak normalized pressure amplitudes obtained from the pressure transducers at OP0 are similar and close to 0.08. In addition, a small variation is observed among the values obtained at the operating points located on the cam curve. This proves that the turbine operation on the cam-curve, within the studied range, produces similar pressure fluctuations on the runner blade.

**Figure 11:**
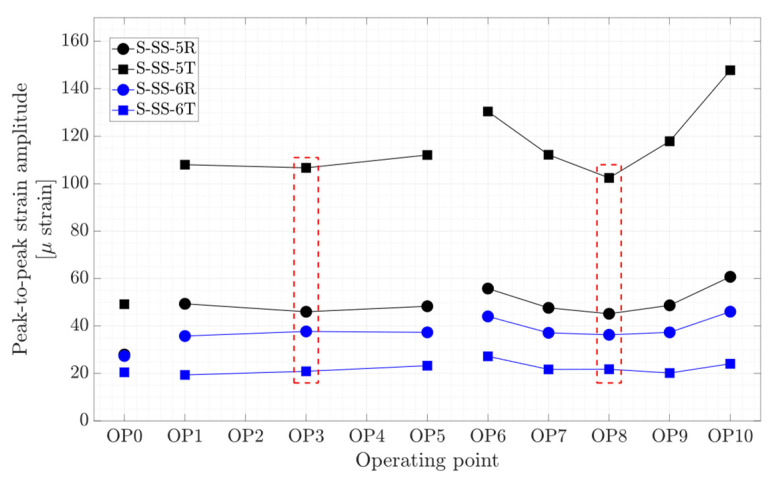
Peak-to-peak strain amplitude obtained on the runner blade at OP0–OP10; red dashed-line boxes indicate the on-cam operating points.

Figure 11 shows the peak-to-peak strain amplitude obtained on the runner blade at four locations close to the blade hub and trailing edge in the radial and tangential directions. The results obtained for OP2 and OP4 are not reported because the strain gages stopped functioning during these operating points. Higher peak-to-peak strain amplitude (100–150 μ strain) is obtained at the location close to the blade hub in the tangential direction, namely S-SS-5T. The minimum pressure and strain fluctuations on the runner blade at each propeller curve correspond to the BEP of that propeller curve, specified by red dashed-line boxes in Figure 10 and Figure 11.

**Figure 12:**
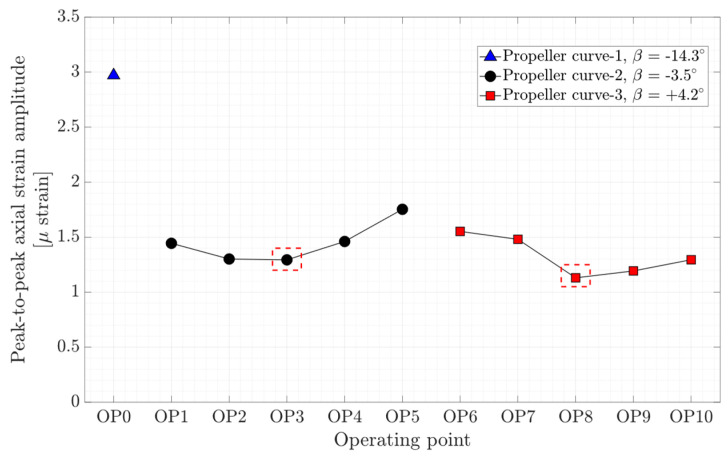
Peak-to-peak amplitude of axial strain obtained on the shaft at OP0–OP10 red dashed-line boxes indicate the on-cam operating points.

**Figure 13:**
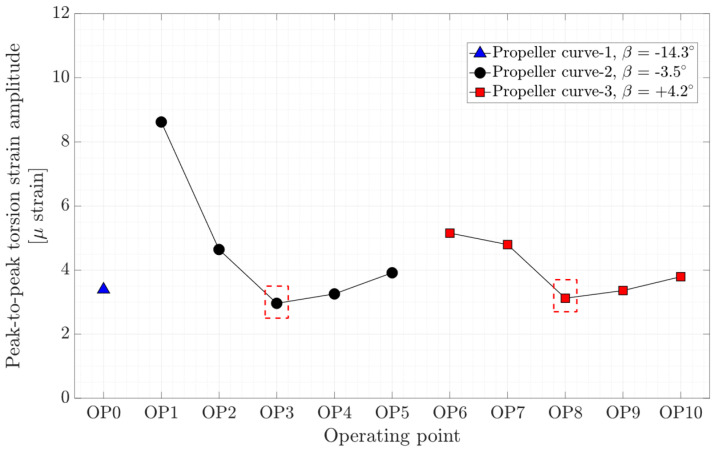
Peak-to-peak amplitude of torsion strain obtained on the shaft at OP0–OP10; red dashed-line boxes indicate the on-cam operating points.

**Figure 14:**
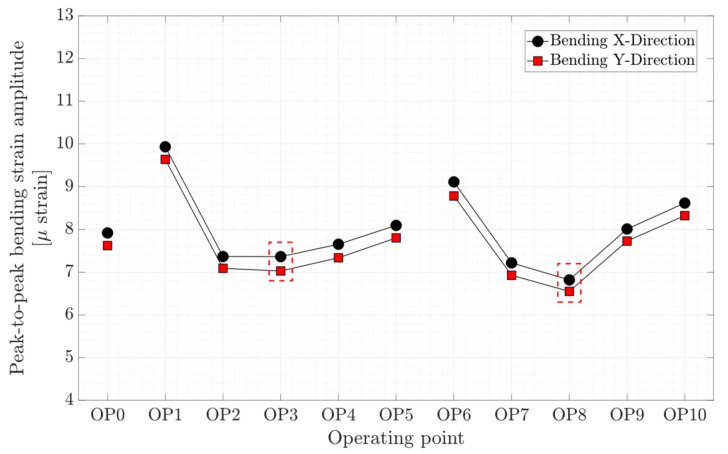
Peak-to-peak amplitude of bending strain obtained on the shaft at OP0–OP10; red dashed-line boxes indicate the on-cam operating points.

The axial, torsion, and bending peak-to-peak strain amplitude at different operating points are presented in Figure 12, Figure 13, and Figure 14, respectively. Local minimums are observed in the figures for each propeller curve occurring at OP3 and OP8. This result shows that the operating points that have minimum peak-to-peak strain amplitude on the shaft and peak-to-peak pressure and strain amplitude on the blade are identical. Despite a similar axial strain magnitude at OP0, OP3, and OP8, the peak-to-peak axial strain amplitude at OP0 is relatively large compare to other operating points. OP0 correspond to a deep part load where the guide vane opening is small. A high swirl is generated that may cause instability leading to axial flow movement. Later in the spectral analysis, the presence of a dominant low-frequency asynchronous mode is detected at this operating point.

**Figure 15:**
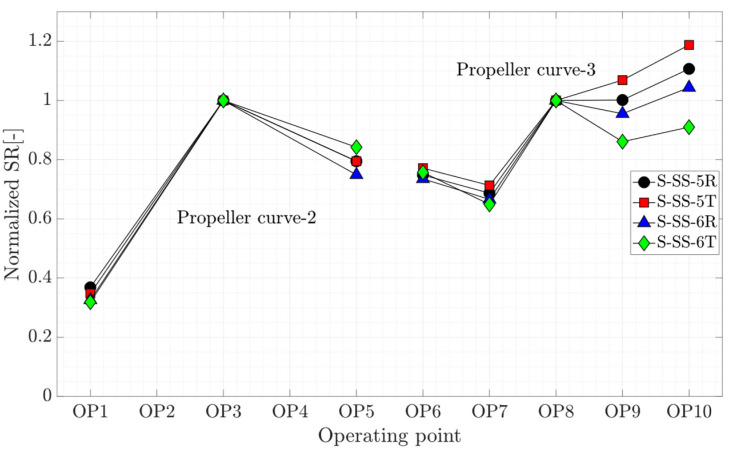
SR variation at OP0–OP10.

As previously mentioned, the main purpose of this project is to find a possible relation between the measurements on the shaft and the runner blade. A non-dimensional number (SR, strain ratio) is defined as follow:

(5) $$SR=\frac{\Delta S_{blade}}{\Delta S_{torsion}}$$

where ΔS is the peak-to-peak strain amplitude. The subscript blade and torsion denote the strain gage on the blade and torsion strain gage on the shaft, respectively. This number is used to assess the ratio of peak-to-peak strain amplitude obtained on the runner blade and torsion strain obtained on the shaft. Figure 15 presents the SR number variation normalized by the SR value of the propeller curve’s BEP for four strain gages installed on the runner blade. Although a different trend is observed for each propeller curve, the SR variation of four strain gages is similar except for OP9 and OP10, which are high load operations. This shows that the ratio of peak-to-peak strain amplitude on the runner blade at two different locations for different operating points is similar.

**Figure 16:**
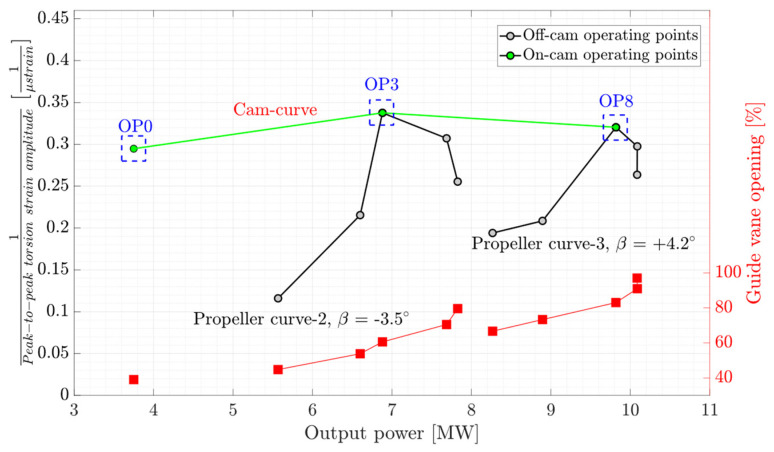
Inverse of peak-to-peak torsion strain amplitude obtained on the shaft as a function of turbine output power. The red lines correspond to guide vane opening. The green line is a hypothetical cam-curve and blue dashed-line boxes indicate the on-cam operating points.

Besides the strain correlation between the blade and shaft measurements, torsion measurement on the shaft showed to be a potential candidate for index-testing in Kaplan turbines. Figure 16 presents the variation of the inverse of the peak-to-peak torsion strain amplitude obtained on the shaft as a function of turbine output power. The right axis of the figure shows the variation of the guide vane opening. A hypothetical line specified by a green color represents the cam-curve. The operating points specified with the blue dashed-line boxes are assumed to be the BEP of the propeller curves and gray circles show the off-cam operating points. A local maximum is observed for each propeller curve representing the BEP of the propeller curve.

### 4.3. Spectral Analysis

**Figure 17:**
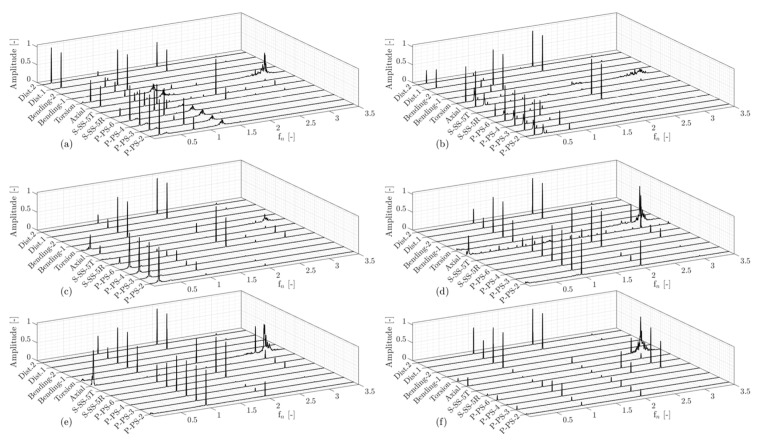
Amplitude spectra of pressure, strain, and displacement data obtained on the runner blade and shaft at low-frequency region; (**a**) OP6, (**b**) OP7, (**c**) OP8, (**d**) OP9, (**e**) OP10, and (**f**) OP0. ‘Dist.1′ and ‘Dist.2′ denote the first and second proximity probes.

Spectral analysis of the data obtained on the runner blade and shaft at different steady-state operating conditions was performed. Figure 17 presents the amplitude spectra of six operating points (OP0 and OP6–OP10). As previously mentioned, the operating points OP6 and OP7 are considered part load operations; two dominant frequencies, the synchronous mode ($f_{n}=0.2$) and asynchronous mode ($f_{n}=0.8$) are clearly observed in different transducer data, see Figure 17a,b. Part load operations can be observed in a Kaplan turbine while operating in on-cam and off-cam mode at different combinations of the runner blades pitch angle and guide vanes opening. Therefore, various intensities and frequencies can be obtained at different part load operating points due to the different swirl numbers. The evolution of RVR modes in model and prototype Kaplan turbines has been investigated during a load increase and a load reduction [19,22]. It was shown that the synchronous component lasts longer during the load increase and appears before the asynchronous component during the load reduction. This can be observed in Figure 17c and where only the synchronous component ($f_{n}=0.2$) is obtained at OP8 in which the discharge is higher than OP7. Moreover, the frequencies of RVR modes change during a load variation. Therefore, different synchronous and asynchronous frequencies are obtained at different part load operating points ($f_{n}=0.18$ and $f_{n}=0.82$ at OP1 and $f_{n}=0.2$ and $f_{n}=0.8$ at OP6). The synchronous mode is seen in the pressure and strain data obtained on the runner blade as well as the axial and torsion strain acquired on the shaft. This component propagates through the entire hydraulic circuit and the runner blades experience a bending strain that can be detected by the strain gages on the blade in the radial direction. The strain gage S-SS-5R installed close to the blade hub in the radial direction shows a clear dominant frequency of this phenomenon, see Figure 17a–c. However, the strain gage installed in the tangential direction does not experience this component. In addition, the second harmonic of the synchronous mode is detected at OP7. The asynchronous mode is observed in all the transducers. This component is caused by the pressure fluctuations due to the rotation of the pressure field with the vortex core. By further increasing the discharge at OP9, the vortex rope frequencies disappear, and only the runner frequency ($f_{n}=1$), and its second harmonic remain in the frequency spectrum of the pressure data. A dominant normalized frequency of $f_{n}=3.15$, which could be related to the shaft torsional mode, is only observed in the torsion strain on the shaft at different operating points. A dominant frequency of $f_{n}=0.07$ is also observed in Figure 17f, which is for deep part load operation (OP0). A dominant frequency in the same range was observed in a load increase from speed-no-load in a Kaplan and propeller turbine [20,23]. This frequency, which could be related to the vortical flow structures in the vaneless space, occurred at speed-no-load operation and moved below the runner by increasing the discharge. It was previously found that the variation of the dominant frequency related to vortical flow structures ($f_{n}=0.93$) was accompanied by the variation of the low frequency ($f_{n}=0.12$) at the same time period but in the opposite direction [20].

**Figure 18:**
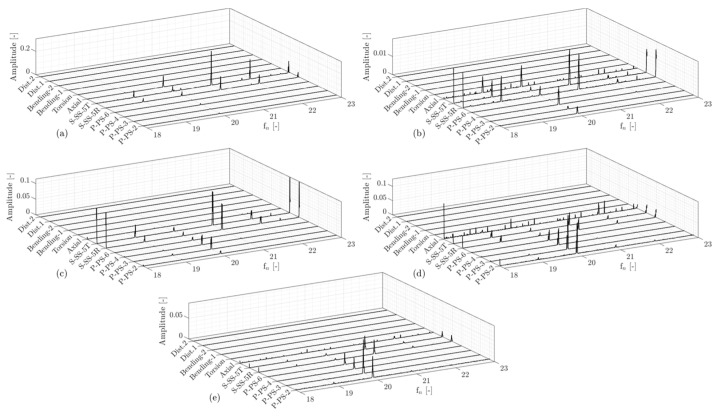
Amplitude spectra of pressure, strain, and displacement data obtained on the runner blade and shaft at high-frequency region; (**a**) OP6, (**b**) OP7, (**c**) OP8, (**d**) OP9, and (**e**) OP10. ‘Dist.1′ and ‘Dist.2′ denote the first and second proximity probes.

The amplitude spectra of the operating points on the propeller curve-3 (OP6-OP10) in the high-frequency region is presented in Figure 18. Guide vane passing frequency ($f_{n}=20$) is observed in the pressure and strain data obtained on the blade. However, it is only detected in the axial strain measurements on the shaft. This proves that a signature of every phenomenon observed in the data obtained on the runner can also be captured in the data on the shaft.

**Figure 19:**
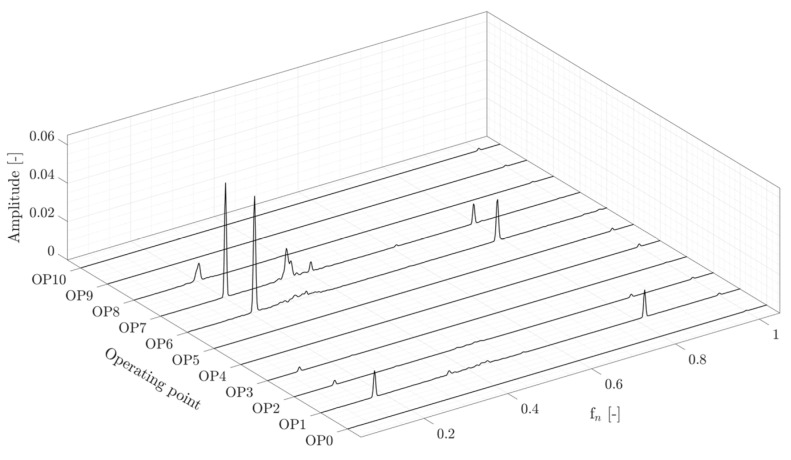
Amplitude spectra of pressure transducer P-PS-3 at different operating points in the RVR frequency range.

It is earlier mentioned that although OP1, OP2, OP6, and OP7 are considered part load operating points, the pressure fluctuation on the runner blade at OP6 and OP7 is higher than that of at OP1 and OP2. Figure 19 presents the amplitude spectra of the pressure transducer P-PS-3 at different operating points. It is observed that the amplitude of the asynchronous component at OP6 and OP7 is four times larger than that of at OP1. Part load operation in higher guide vane opening and runner blades pitch angle (OP6 and OP7) results in a vortex rope with a predominant axial flux that creates a stronger synchronous component with high-pressure fluctuations on the runner. A similar phenomenon has also been observed in a swirl generator by comparing the flow structure after the runner at three different blade angles [24].

**Figure 20:**
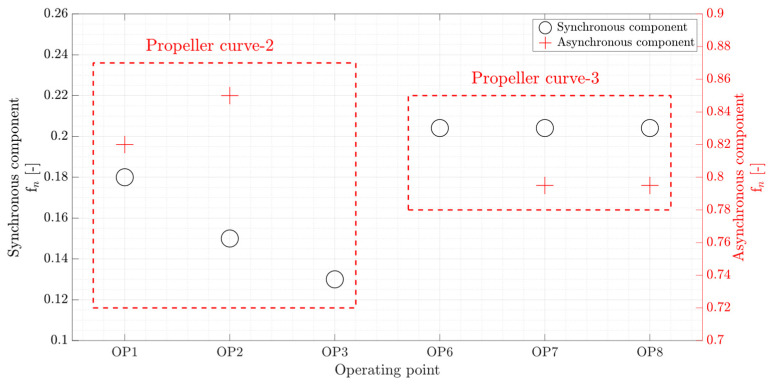
Variation of RVR frequencies obtained from amplitude spectra of pressure transducer P-PS-3 at six operating points.

The vortex rope synchronous and asynchronous frequency variation due to a load change in the part load operation range has been investigated in Francis and Kaplan turbines [19,25]. Figure 20 shows the variation of RVR frequencies at six operating points in the range of part load operation for propeller curve-2 and propeller curve-3. As the discharge increases from OP1 to OP3, the synchronous frequency decreases, and the asynchronous frequency increases. However, the RVR frequencies remain unchanged at OP6, OP7, and OP8 in which the runner blades pitch angle is higher. This shows that the vortex rope structure depends on the discharge and runner blades pitch angle in Kaplan turbines.

All the hydraulic phenomena cannot be detected with a single transducer installed on a hydraulic turbine. However, the spectral analysis showed that different hydraulic phenomena within a range of $f_{n}=0\;\sim \;20$ can be captured by measurements on the turbine shaft. Table 3 presents a summary of the hydraulic phenomena observed at each transducer in the rotating and stationary frame of reference. All the pressure transducers installed on the runner blade detected similar hydraulic phenomena. Therefore, the pressure transducer P-PS-2 is only presented. The results show that axial strain measurements on the shaft could be a suitable alternative to detect the flow phenomena in the turbine chamber.

## 5. Conclusions

A measurement methodology for predicting the hydraulic phenomena and load fluctuation in a Kaplan turbine is introduced. This is based on the instantaneous measurements on the runner blade and the shaft. Pressure and strain measurements on the runner blade, strain measurements on the shaft, and shaft displacement measurements were performed at eleven steady-state operating points. One operating point was at deep part load operation and ten operating points were located on two propeller curves.

Through the analysis of experimental data, any possible correlation between the load fluctuations on the runner and the shaft of a prototype Kaplan turbine was investigated. The results highlighted that the operating points that have minimum peak-to-peak strain and pressure amplitude on the blade and peak-to-peak strain amplitude on the shaft are identical and correspond to the assumed BEPs of the propeller curves. The axial strain magnitude on the shaft at different on-cam operating points is rather constant, although different guide vane openings and runner blades pitch angles were assigned to those operating points. Similarly, a small variation of the peak-to-peak pressure amplitude on the blade is observed at the operating points located on the cam curve. Moreover, the results demonstrated that the ratio of peak-to-peak strain amplitude on the runner blade at two different locations for different operating points is similar. This result could also be detected by the torsion measurements on the shaft.

Different flow phenomena in a hydraulic turbine could be captured by different transducers installed at various locations in a hydraulic turbine. The results showed that the hydraulic phenomena with a frequency range of $f_{n}=0\;\sim \;20$ in the studied operating points could be captured by the measurement on the turbine shaft. Among the measurement data obtained on the shaft, the axial strain measurements captured all the dominant frequencies observed on the runner blade. Moreover, the torsion strain measurement on the shaft seems to have a promising prospect in the index testing of Kaplan turbines.

The results achieved in this paper allows for the interpretation of predicting flow conditions by performing indirect measurements, e.g., turbine shaft measurements. This can guarantee a safe operation of hydraulic turbines while operating at high load fluctuation zones. In particular, this could introduce a low-cost methodology to update the index-test results for Kaplan turbines while the unit is operating. In the literature, any possibility of using this methodology has not been investigated for other types of hydraulic turbines. To validate the methodology in this respect for Kaplan and Francis turbines, further reduced scale model and prototype turbine measurements should be performed.

## Figures and Tables

**Table 1:** Nominal operating parameters of the Porjus U9 prototype Kaplan turbine.

Parameter [Unit]	Value
Head [m]	55.5
Power [MW]	10
Discharge $\left[m^{3}s^{-1}\right]$	20
Rotational speed [RPM]	600

**Table 2:** Accuracy and uncertainties of the pressure transducers and strain gages installed on the turbine runner blade and shaft.

Transducer Name	Accuracy	Maximum Uncertainty ^1^ (%)	Transducer Type and Position of Installation
P-PS-2	± 0.1% full scale output (FSO) best fit straight line (BFSL) (typical), ± 0.5% FSO (maximum)	0.71	Pressure transducers installed on the runner blade (Kulite LL-080 series)
P-PS-3		1.63	
P-PS-4		0.91	
P-PS-6		1.11	
S-SS-5R	± 0.1% FSO BFSL (typ.), ± 0.5% FSO (max.)	1.80	Strain gages installed on the runner blade (K-LY41-6/350-3-2M manufactures by HBM)
S-SS-5T		2.06	
S-SS-6R		3.96	
S-SS-6T		2.62	
Torsion strain gage	± 1% gage factor tolerance ± 0.35% resistance tolerance 0.3% transverse sensitivity	3.42	Strain gages installed on the shaft (K-XY41-6/350-3-2M manufactures by HBM)
Axial strain gage 1	± 1% gage factor tolerance ± 0.35% resistance tolerance 0.1% Transverse Sensitivity	5.78	Strain gages installed on the runner blade (K-LY41-6/350-3-2M manufactures by HBM)
Axial strain gage 2		7.79	
Axial strain gage 3		6.00	
Axial strain gage 4		7.95	
Proximity probe 1	± 0.01 mm Repeat accuracy (constant temperature)	2.50	DW-AD-509-M12 manufactured by Contrinex
Proximity probe 2		3.60	

**^1^** Maximum uncertainty of the measured value.

**Table 3:** A summary of the spectral analysis results regarding hydraulic phenomena detection with transducers installed in the rotating and stationary frame of reference; ✓, -, and × denote ‘detected’, ‘not detected’, and ‘the transducer is not applicable’, respectively.

Phenomenon	P-PS-2	S-SS-5R	S-SS-5T	Axial Strain	Torsion Strain	Bending Strain	Proximity Probe
RVR-Synchronous mode	✓	✓	-	✓	✓	-	×
RVR-Synchronous mode*2	✓	✓	-	✓	✓	-	×
RVR-Asynchronous mode	✓	✓	✓	✓	✓	✓	✓
$f_{n}=$ 1	✓	✓	✓	✓	✓	✓	✓
$f_{n}=$ 2	✓	✓	✓	✓	✓	-	✓
$f_{n}=$ 20	✓	✓	✓	✓	-	-	-
